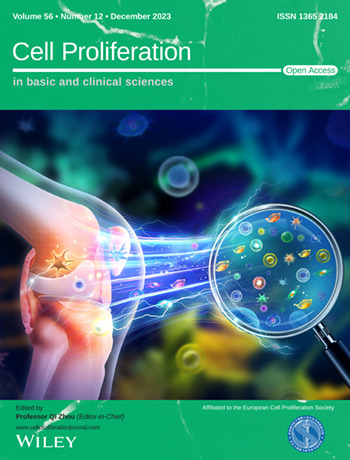# Featured Cover

**DOI:** 10.1111/cpr.13584

**Published:** 2023-12-02

**Authors:** Yuyuan Gu, Yan Hu, Hao Zhang, Sicheng Wang, Ke Xu, Jiacan Su

## Abstract

The cover image is based on the Review *Single‐cell RNA sequencing in osteoarthritis* by Yuyuan Gu et al., https://doi.org/10.1111/cpr.13517.